# The Dowsing Radiant Heat Baths

**Published:** 1902-06-21

**Authors:** 


### THE DOWSING RADIANT HEAT BATHS.

There is no subject in the whole domain of therapeutics which presents such a fascinating and all-absorbing interest, not only for ultra-scientific specialists but also for the man with intelligence in the street, as that of electricity. The applications and possibilities of this subtle form of energy in mechanical processes are still only in the early stages of development, while our knowledge of the action of electrical forces in the case of living organisms is still entirely empirical. What do we know, for instance, of the physiological chaos which reigns in the human body under the atmospheric and electrical disturbances of a thunder storm? We know that we are oppressed with a sense of impending evil; we feel that all our neuralgic tendencies are being aroused to activity, but we know nothing of the electrical exchanges between our bodies and the atmosphere which surrounds us. With these unruly forces of nature, which can be operative of so much evil, there lurk equal potentialities for good. Those of us who have followed the progress of the therapeutic measures in which electricity is employed, cannot help realising that within the last few years great advances have been made in their practical applications. It was only a few years ago that in his capacity of electrical engineer, Mr. Dowsing turned his attention to the utilisation of the radiant heat of electric lamps as a means of treatment, and now at the present time we understand that there are 70 establishments in different parts of the country in which the apparatus which bears his name is being systematically used. The practical utility of the Dowsing radiant heat baths has been proved beyond a doubt, and its therapeutic value is unquestionable. The method combines the advantages which accrue from the local application of heat, combined with the little understood therapeutic action of light. The source of the energy which reaches the patient in the form of luminous radiant heat is that of special electric lamps, which are fixed in burnished reflectors of suitable design, and which are easily adjustable for the concentration of the rays upon any particular area of the body, or, if so desired, for their equable distribution over its entire surface. The complete Dowsing bath consists of a bed fitted with an asbestos-lined mattress and blanket, and having five reflectors, each containing two Dowsing lamps with a rheostat for regulating the current. The patient strips himself and lies down upon the mattress, being covered with an asbestos-lined blanket. The head is the only part which is exposed, and consequently by this arrangement he is enabled to breathe the normal atmosphere of the room. The current is then turned on, and by means of the rheostat the enclosed space is brought to the required temperature. The luminous heat rays are of such intensity that the temperature taken near the surface of the skin by a special thermometer may reach 400° F., or even more. How such temperatures can be tolerated by the human body, without damage resulting, is one of the curiosities of physiology. It is clear that if the surrounding air were saturated with moisture it would be impossible to submit the body to a temperature anything approaching this degree, and one of the advantages of the Dowsing system is that the air which surrounds the patient is constantly changing and never becomes highly charged with water vapour. Without entering further into the practical details of the method of heating the body by luminous heat rays, it may be of advantage to shortly summarise the physiological effect of such rays upon the body. In the first place, there is hyperæmia of the skin and abundant perspiration; there is an increased elimination of carbonic acid by the lungs and of urea by the kidneys; the pulse is accelerated and the frequency and amplitude of the respiratory movements are increased. These physiological effects are concomitant with, or perhaps the result of, a general augmentation of the metabolic or nutritional processes throughout the body. Undoubtedly these changes go far to explain the efficacy of this form of surface stimulation in cases of malnutrition and metabolic inadequacy, such as in anæmia, gout, rheumatism, and nervous debility. The alleviation of pain and inflammatory conditions is probably due to the same influence, but is not so easy to explain. The Dowsing system can be applied in the patient's own home, though more satisfactorily in the institutions which are specially equipped for the purpose. At the chief of these, which is located in York Place, Baker Street, in addition to the radiant heat appliances, other methods of physical therapeutics are available, such as hydro-electric, vibratory and high frequency apparatus; the light treatment for lupus cases can also be applied by means of a greatly simplified apparatus.

